# Decrease in hepatitis B prevalence among blood donors in Central-West Brazil

**DOI:** 10.1186/1678-9199-19-7

**Published:** 2013-04-08

**Authors:** Andréa de Siqueira Campos Lindenberg, Ana Rita Coimbra Motta-Castro, Marco Antonio Puga, Tayana Serpa Ortiz Tanaka, Marina Sawada Torres, Sonia Maria Fernandes-Fitts, Rivaldo Venancio Cunha

**Affiliations:** 1 Federal University of Mato Grosso do Sul, Campo Grande, Mato Grosso do Sul State, Brazil; 2 Mato Grosso do Sul Hematology Center, Campo Grande, Mato Grosso do Sul State, Brazil

**Keywords:** Hepatitis B virus, Prevalence, Blood donors

## Abstract

**Background:**

The aim of the present study was to estimate hepatitis B virus seroprevalence among first-time blood donors in the city of Campo Grande, Mato Grosso do Sul State, in the central-western region of Brazil.

**Findings:**

A retrospective analysis of first-time voluntary blood donor records, from January 2010 to December 2010, was conducted at the Hematology Center of Mato Grosso do Sul. The prevalence of the HBsAg and anti-HBc serological markers and their respective 95% confidence intervals were calculated. Chi-square analysis was performed between the seroprevalence previously found in 2001 and the one determined by the current study. Results were considered statistically significant if *p* < 0.05. Among 8,840 subjects, 269 (3.04%, 95% CI: 2.7-3.4) were positive for HBV markers. The prevalence rate of HBsAg was 0.19% (95% CI: 0.1-0.3) and anti-HBc alone was 2.85% (95% CI: 2.5-3.2).

**Conclusions:**

There was no statistically significant difference regarding gender. However, an important association was observed between HBV infection and older age (*p* < 0.01). The seroprevalence of HBV infection in first-time blood donors diminished from 2001 to 2010 (*p* < 0.01). Such decrease suggests an improvement in the recruitment of safe donors, the positive impact of vaccination programs and the decreasing of HBV infection prevalence in the general population.

## Findings

Hepatitis B is one of the most common infectious diseases throughout the globe and has infected two billion people, including an estimated 350 million chronically infected cases [1,2].

Parenteral exposure has been shown to be associated with hepatitis B virus (HBV) infection. Blood transfusions carry the risk of transfusion-transmitted infections such as hepatitis B and C. In order to measure their severity, the World Health Organization has recommended a pre-transfusion blood test. The residual risk of infection from HBV is higher than that of hepatitis C virus (HCV) in non-endemic countries [3].

In 1971, hepatitis B surface antigen (HBsAg) test was introduced for screening blood donors, and after 1986, antibody tests to hepatitis B core antigen (anti-HBc) were implemented to further reduce the risk of infectious agents in the blood supply [4].

The prevalence of infection in the general population varies in different regions of the world. Although Brazil is considered a low endemic country, there are areas that are highly endemic for HBV in the Amazon region [5]. In Brazil, among blood donors, the prevalence ranges from 0.3% to 1.5% for HBsAg and from 3.7% to 11.1% for anti-HBc [4,6,7].

The aim of the present study was to estimate the seroprevalence of HBV infection among first-time blood donors in the city of Campo Grande, the capital city of the state of Mato Grosso do Sul, in the central-western region of Brazil.

A retrospective analysis of blood donor data from January 2010 to December 2010 was conducted in the Hematology Center of Mato Grosso do Sul (HEMOSUL). People who attended the center voluntarily for their first blood donation were selected by the standard criteria for healthy blood donors after answering a standardized questionnaire to evaluate epidemiological data. First-time blood donor was defined as a donor who donated for the first and only time. The protocol used in the present study was approved by the Ethics Committee of the Federal University of Mato Grosso do Sul.

Blood donor samples were screened for the presence of HBsAg and total anti-HBc by enzyme-linked immunosorbent assays (ELISA) (bioMérieux, Netherlands). Positive samples were retested using the same serological assay.

The prevalence of the HBsAg and anti-HBc serological markers and their respective 95% confidence intervals (95% CI) were determined. Chi-square analysis was performed between the seroprevalence published in 2001 and seroprevalence found in this study (2010) and was considered statistically significant if *p* < 0.05.

A total of 8,840 first-time blood donors aged between 18 to 65 years, who were considered healthy individuals and eligible for blood donation, participated in this study. The majority of individuals were males (60.6%, n = 5,358) between 18 and 39 years old (81.2%, n = 7,074), representing typical characteristics of blood donors in Brazil. Among the 8,840 subjects, 269 (3.04%, 95% CI: 2.7-3.4) were positive for HBV markers. The prevalence rate of HBsAg was 0.19% (95% CI: 0.1-0.3) and anti-HBc alone was 2.85% (95% CI: 2.5-3.2). There was no statistically significant difference with respect to gender. A significant association was observed between HBV infection and older age (*p* < 0.01) (Table 1).

**Table 1:** Prevalence of hepatitis B viral infection among first-time blood donors by gender and age in Campo Grande, Brazil, 2010

**Variable**	**HBV**	**%**	***Odds ratio***	***p***
	**Positive/Total**		**(95% CI)**	
**Gender**				
Female	107/3,482	3.07	1.0	
Male	162/5,358	3.02	0.98 (0.76-1.27)	0.94
**Age (years)**				
18-28	99/4,990	1.98	1.0	
29-39	75/2,258	3.32	1.70 (1.24-2.33)	< 0.01
40-50	61/1,130	5.40	2.82 (2.01-3.95)	< 0.01
51-66	34/462	7.36	3.92 (2.57-5.97)	< 0.01

As shown in Table 2, the seroprevalence of HBV infection in first-time blood donors was reduced significantly from 2001 to 2010 (*p* < 0.01).

**Table 2:** Difference in the prevalence of hepatitis B viral infection among first-time blood donors between 2001 and 2010, Campo Grande, Brazil

**Variable**	**HBV**	**%**	***Odds ratio***	***p***
**Year**	**Positive/Total**		**(95% CI)**	
2001*	52/552	9.42	3.31 (2.40-4.57)	< 0.01
2010	269/8,840	3.04		

*Aguiar *et al*. [9].

Despite the decreasing of the global HBV infection, the use of serological markers for blood donor screenings is still important, since HBV remains a great risk for blood transfusion patients [3,8]. In this study, the prevalence of HBV infection was 3.04% (95% CI: 2.7-3.4) and 0.19% (95% CI: 0.1-0.3) for total anti-HBc and HBsAg, respectively, suggesting the presence of HBV infection in first-time blood donors. However, this is a significant drop compared to 9.42% for total anti-HBc and 0.72% for HBsAg prevalence rate reported by Aguiar *et al*. [9].

Considering that HBV infection prevalence in blood donors depends on several factors, including the prevalence of the virus in the general population and the sensitivity of screening tests, it is reasonable to assume that the observed decline may have been due to the implementation of safety measures. Particularly because the screening tests used over the past decade were of similar sensitivity and performed in the same laboratory (Hemorrede Laboratory of Mato Grosso do Sul) with similar technical capabilities.

The cause of such declined rates is multifactorial and most likely due to the increase in vaccination programs conducted in the general population, implementation of a national strategy for blood safety and better data quality. Although over the past few years several studies have shown a decreasing trend of HBV seroprevalence among blood donors, others have found opposite results [10-16]. According to Niederhauser [17], immunization campaigns are efficient in reducing the risk of transfusion-transmitted HBV.

During the last four decades, blood donor profiles have changed as a result of the implementation of HBV screening, which has steadily reduced the risk of transmitting the virus through blood transfusion [18]. Moreover, there is a cumulative effect of increasing public health awareness [19]. In the population-based multicentric survey of hepatitis B infection in central-west Brazil, the prevalence of HBV rates classify the region as low endemic, rather than intermediate endemic as defined by previous studies [5]. Such drop may be the explanation for the lower seroprevalence of HBV infection among blood donors than found by Aguiar *et al*. [9].

Significant increase in the seroprevalence of HBV was observed among older people. The lower prevalence among donors under 30 years old could be associated with hepatitis B vaccination program, which was initiated at the beginning of the 1990s in Brazil. Moreover, higher prevalence among older donors indicates a longer time of viral exposure, as corroborated by O’Brien *et al*. [20], Nkrumah *et al*. [13] and Seo *et al*. [21].

Statistical analysis showed no significant difference in HBV infection prevalence according to gender. This finding is similar to those found by Japhet *et al*. [22], Nascimento *et al*. [6] and Ataallah *et al*. [23].

Our results demonstrate the importance of updated information on HBV prevalence. The significant decrease of HBV infection between 2001 and 2010 among first-time blood donors suggests an improvement in the recruitment of safer donors, the positive impact of vaccination programs and the drop of HBV infection prevalence in the general population. This is a welcoming result since the safety of blood products also brings benefits to the general population and guides new prevention strategies.

### Ethics committee approval

The present study was approved by the Ethics Committee of the Federal University of Mato Grosso do Sul (n. 1721/2010).

## Competing interests

The authors declare that there are no competing interests.

## Authors’ contributions

ASCL, ARCMC, SMFF and RVC designed the study. MAP and TSOT did most of the laboratory work. MST, SMFF and ASCL provided and checked the clinical data for patients. ASCL, ARCMC and SMFF wrote the manuscript. All authors reviewed the draft and approved the final version.
